# Microstructures and Properties Investigation on DP980 Dual-Phase Steel CMT + P Welded Joints

**DOI:** 10.3390/ma15175880

**Published:** 2022-08-25

**Authors:** Yan Liu, Zhaozhen Liu, Yongman Chen, Chunlin He, Ao Liu, Xiaoang Liu

**Affiliations:** 1 Liaoning Provincial Key Laboratory of Advanced Material Preparation Technology, Shenyang University, Shenyang 110044, China; 2 School of Mechanical Engineering, Shenyang University, Shenyang 110044, China

**Keywords:** DP980 dual-phase steel, CMT + P welding technology, microstructures, mechanical property, softened zone

## Abstract

The increasing demand for the lightweight production of advanced high-strength steel puts forward higher requirements for the quality of welded joint forming. The common CMT welding process has certain limitations and is difficult to meet the needs of lightweight manufacturing. In this study, the CMT + P welding technology was used to weld the DP980 dual-phase steel plate with 1.2 mm thickness. The ER120S-G welding wire was used as the filling material to conduct a 70° angle lap welding experiment. The effects of wire feeding speed (3 m/min~5 m/min) and welding speed (350 mm/min~600 mm/min) on the forming, microstructures, and mechanical properties of DP980 dual-phase steel welded joints were analyzed. The results show that the CMT + P welding process can produce lap weldments with good formability and properties. The welded joints can be divided into the weld zone, the HAZ, and the base metal zone, where the HAZ can be divided into the coarse-grained zone and the softened zone. The role of the elements Ni and Si is to promote the production of martensite and to increase the ferrite strength in welded joints. As the wire feeding speed increases, the grain size of the coarse grain zone in the HAZ increases from 31.90 μm to 50.93 μm; while the welding speed increases, the grain size of the coarse grain zone decreases from 45.48 μm to 35.73 μm. The average microhardness of the weld zone is 420 HV. In contrast, the average microhardness of the softening zone in HAZ is reduced to 250 HV. When the wire feeding speed is 4 m/min and the welding speed is 550 mm/min, the tensile properties of the weldment are optimal, its tensile strength can reach 973 MPa, and the tensile fracture is ductile fracture.

## 1. Introduction

To accomplish the goal of green development, energy conservation, and emission reduction, lightweight components are increasingly used in rail transit and other manufacturing fields [1]. As a new generation of advanced high-strength steel, dual-phase steel has higher strength and better toughness than traditional high-strength steel [2,3,4,5]. In the field of automobile manufacturing, dual-phase steel is used instead of conventional high-strength steel to manufacture automobile chassis, brackets, and control arms, which not only makes the automobile have a stronger anti-impact ability but also reduces the weight of the automobile by 10–20%. Therefore, dual-phase steel is one of the ideal structural materials in the modern industry [6,7,8]. Although dual-phase steel has good welding performance, the welded components with good forming effect cannot be produced by using the traditional tungsten inert gas (TIG) and melt inert gas (MIG) welding process. As a new and improved MIG connection technology, cold metal transfer (CMT) has the welding characteristics of low heat input and no spatter after welding. It can solve some welding defects in traditional fusion welding and has a broad application prospect in the field of dual-phase steel sheet welding [9,10,11,12]. Therefore, the combination of advanced welding technology and high-strength dual-phase steel can solve the problems of low welding quality and poor forming in practical production. It can further promote the broad application of dual-phase steel in the modern automobile manufacturing industry.

Researchers at home and abroad have conducted some related investigations on the welding process of dual-phase steel over the past few years. Cui et al. [13] used resistance spot welding technology to conduct welding experiments on DP780 dual-phase steel with 1.6 mm thickness and studied the failure behavior of welded joints. Finally, the tensile part broke under 21.08 kN load. Deng et al. [14] conducted welding experiments on DP800 dual-phase steel with 1.2 mm thickness by laser welding. The results showed that the microhardness of the weldments varied from 221 HV to 243 HV. Sezgin et al. [15] used electron beam welding technology to weld DP1000 dual-phase steel with 1.2 mm thickness. They found that the average tensile strength of the weldments was 656 MPa. In addition, Huang et al. [16] studied the arc behavior of the CMT welding in a unique environment, and the results showed that the welding arc stability of the CMT welding technology was good, and the test data was higher than the standard value, which could reach the operation requirements of the unique environment.

According to the short-circuit transition principle of the CMT, the welding process is characterized by continuous low heat input, while it is only suitable for welding thin plates. If the heat input is increased, the area of the softened zone in the heat-affected zone (HAZ) will be increased, which will affect the service performance of materials. However, reducing the heat input is prone to welding defects such as incomplete fusion and incomplete penetration [17,18,19]. Based on the CMT welding process, the cold metal transfer plus pulse technology (CMT + P) is proposed to improve the narrow control range of the CMT welding heat input [20,21,22]. Pulse can change the current output by accelerating the flow rate of electrons, which makes up for the deficiency of the typical CMT welding process, that is, the small weld depth and only suitable for thin plate welding. It is easy to obtain a welded joint with a relatively large weld depth-to-width ratio [23,24,25,26,27]. In this study, the DP980 dual-phase steel plate with 1.2 mm thickness was welded by the CMT + P welding technology. The effects of wire feeding speed and welding speed on the forming, microstructures, and mechanical properties of DP980 dual-phase steel welded joints were analyzed to provide experimental data and theoretical support for realizing high-quality and high-efficiency welding of dual-phase steel.

## 2. Materials and Methods

In this experiment, the research object is the DP980 dual-phase steel for automobile frame. The size of the test plate is 150 mm × 125 mm × 1.2 mm. ER120S-G welding wire with diameter Φ = 1.0 mm is selected. The chemical compositions of both are shown in Table 1. The experimental protective gasses are CO_2_ and Ar mixed in a ratio of 1:4.

**Figure 1:**
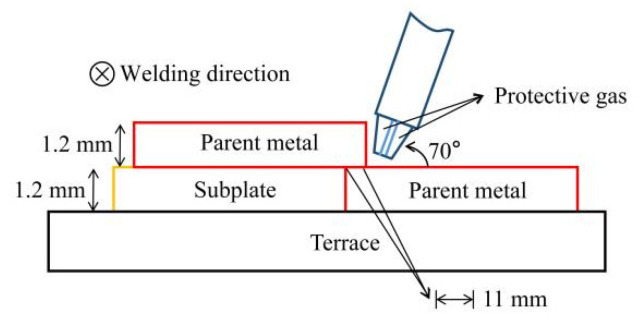
Cross-section view of the CMT + P welding process.

**Figure 2:**
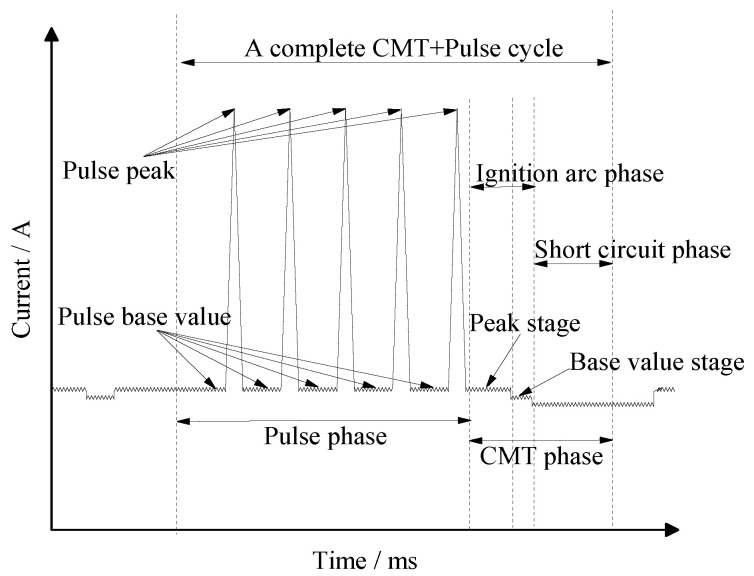
Schematic diagram of the CMT + P welding.

The TPS-3200 CMT welder produced by the Fronius company in Austria was employed as welding equipment. DP980 dual-phase steel was welded by overlapping. The overlapping width of the two plates was 11 mm, the included angle between the welding torch and the parent metal of the workpiece was 70°, and the dry elongation of the welding wire was 10 mm. The schematic diagram of the welding process is shown in Figure 1. Before welding, the basic metal surface of DP980 dual-phase steel was polished with sandpaper to remove the surface oxide film. Then, the base metal was clamped firmly on the welding platform surface, and the CMT + P welding mode was used for the welding experiment on the CMT welder. The working principle of pulse current is shown in Figure 2. The gas flow rate was controlled at 25 L/min. The arclength correction coefficient was 0%. In the CMT + P welding mode, the forming, microstructure, and mechanical properties of welded joints are studied by adjusting the wire feeding speed (3 m/min~5 m/min) and welding speed (350 mm/min~600 mm/min).

**Figure 3:**
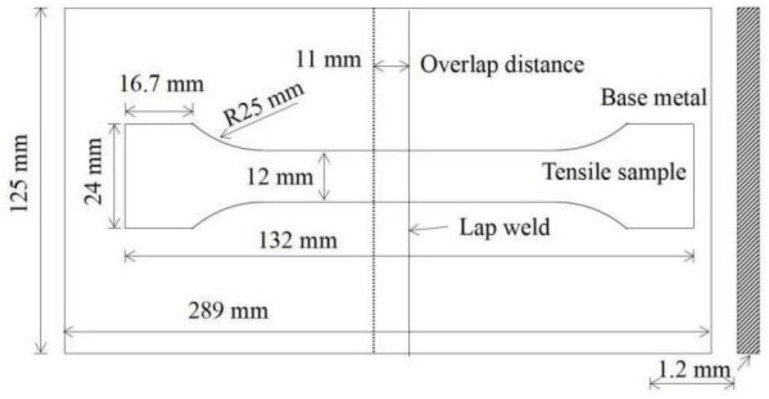
Schematic diagram of tensile sample size.

After welding, the metallographic samples of 30 mm× 10 mm× 1.2 mm were prepared by an electric spark CNC wire-cutting machine and were corroded with 4% nitric acid alcohol reagent after grinding and polishing. The energy dispersive spectrometer (EDS) equipped with a scanning electron microscope (SEM) was used to scan the welded joint area to determine the main elements of the welded joint and analyze the effect of alloying elements in the welding process. The resolution of the SEM is 1.0 nm (15 kv), and the acceleration voltage is 0.5~30 kv. The OM morphology of the welded joint HAZ was observed by the metallographic microscope. The grain size of the coarse-grain zone was measured according to GB/T 6394-2017 “Determination of estimating the average grain size of the metal”. Secondly, the microhardness distribution of welded joints was measured by a digital Vickers hardness testing machine with a load of 200 g and was applied for 15 s. The welded joint area was dotted every 0.3 mm from the position of the weld to the base metal. Three groups of samples were tested, and the microhardness curves were plotted using the Origin mapping software based on the measurement results. Finally, the tensile properties of welded joints were tested by a microcomputer-controlled electronic universal testing machine. The size of the tensile sample was set according to the GB/T 2651-2008 “Tensile test method on welded joints”, as shown in Figure 3. A test plate with an equal thickness was added on both sides of the lapweld, and the tensile test was conducted at a tensile rate of 2 mm/min. The fracture microstructures of the welded joints were observed by the SEM.

Welding line energy is defined as the ratio of welding power to welding speed, which reflects the quantity of heat supplied by the welding energy input of each welding piece within the unit distance. With the change of welding parameters, the welding line energy also changes. The shift in welding line energy will lead to changes in microstructures and mechanical properties in HAZ. Therefore, this paper focuses on the effect of the welding process conditions on the welding line energy and how this change affects the mechanical properties of the welded joints. The calculation formula of welding line energy is:

*Q = UI/V* (1)

where:

*Q*—welding line energy (J/cm),

*U*—welding voltage (V),

*I*—welding current (A),

*V*—welding speed (cm/s).

The depth-to-width ratio can be used to evaluate the effect of weld forming. When applying different welding processes to weld the same material, under the specified weld depth, the larger the weld depth-to-width ratio, the better the welding forming effect. If the weld width is too wide, the HAZ area in the welded joints will increase. It influences the comprehensive performance of the welded structure. In addition, an excessively wide weld will reduce the protective effect of the gas and result in the mixing of harmful elements during the welding process. Therefore, it is of great significance to measure the depth-to-width ratio of the weld in this experiment, and its calculation formula is:

*m = D/W* (2)

where:

*m*—the depth-to-width ratio of the weld,

*D*—weld depth (μm),

*W*—weld width (μm).

## 3. Results

### 3.1. Typical Macromorphology and Element Distribution of Welded Joints

**Figure 4:**
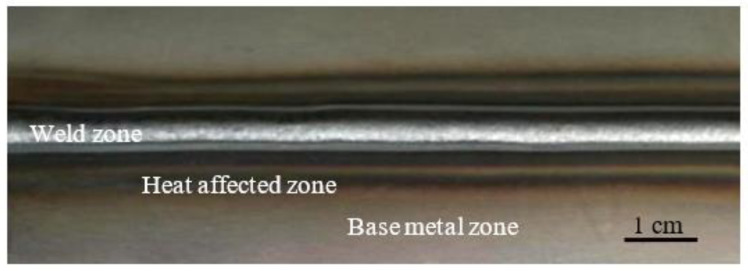
The CMT + P welded joint of DP980 dual-phase steel.

The typical macroscopic morphology of the welded joint fabricated by CMT + P technology is shown in Figure 4. It can be gained that the surface forming effect of the welded joint is well. There are no welding defects such as spatter residue, non-fusion, and arc crater. In Figure 4, the welded joints are divided into weld zone, HAZ, and base metal zone. The HAZ is between the base metal and the weld zone. It is symmetrically distributed at the center of the weld zone. The HAZ close to the fusion line is the weakest area between the welding wire and the base metal. Therefore, the performance of the HAZ determines the overall performance of the component. Starting from the wire feeding speed and welding speed, the paper mainly explored the effects of the CMT + P welding process parameters on the welding forming, microstructures, and properties of the HAZ in DP980 dual-phase steel welded joints.

**Figure 5:**
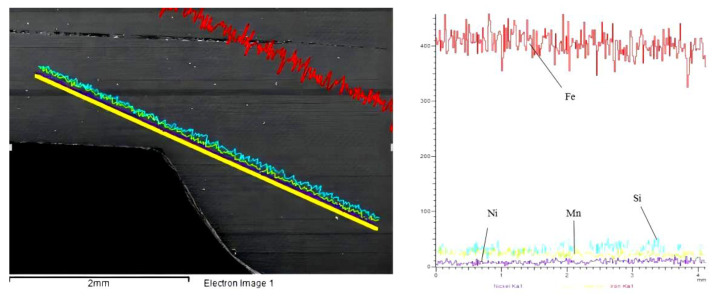
EDS detection results of welded joint.

In the welding process, the effect of alloying elements on the properties of the welded joints is noticeable. Different wire components have different effects on the structure of welded joints [28]. The ER120S-G welding wire is selected as the experimental welding wire after careful consideration. First, the selection of welding wire follows the principle of “equal strength matching”. The tensile strength of the welding wire is 1043 MPa. Secondly, compared with the base metal, the contents of Si and Ni elements are increased in the welding wire. The Si has a more vital deoxidation ability than the Mn, and it is easily soluble in ferrite. Therefore, it can strengthen the toughness of the ferrite [29,30]. The addition of Ni to steel can improve the hardenability of the dual-phase steel and increase its strength. There are two methods to improve the mechanical properties of welded joints. One is to decrease the relative content of the ferrite by adjusting the welding process parameters. Another is to add alloying elements to enhance the toughness of the microstructures. Finally, the content of Mn in the welding wire is reduced. The Mn content should not exceed 1.8% to improve the strength and toughness of the welded joint. The main element compositions of the welded joints were analyzed by the EDS line scanning, and the results are shown in Figure 5. The element of the Si has an excellent toughening effect and low crack sensitivity in welded joints. The boiling point of the Mn is 1962 °C and the general arc welding temperature can reach about 3000 °C, which is easy to cause the evaporation and burning loss of the Mn [31,32]. The element of the Ni can decrease the brittle transition temperature and increase the hardness and strength of welded joints [33,34,35]. Moreover, Ni and Fe belong to an infinite solid solution, which can reduce the content of proeutectoid ferrite to improve the strength of welded joints.

### 3.2. Depth-to-Width Ratio of Welded Joints in Different Welding Parameters

**Figure 6:**
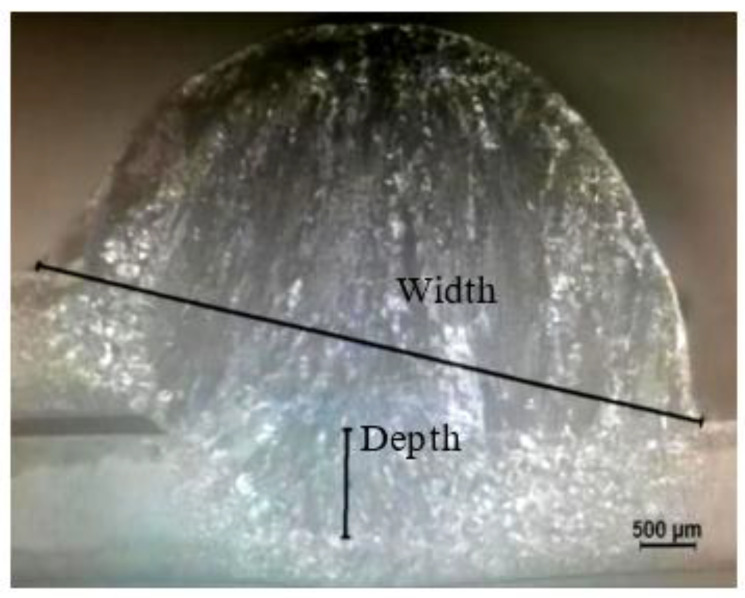
Section morphology of welded joint.

The cross-sectional morphology of the welded joint observed at a 20-fold metallographic microscope magnification is shown in Figure 6. It shows the measuring method of depth and width. The welding torch at a certain angle with the welded is to obtain a welded joint with well formability. After several pre-experiments in the study, the welding torch in the angle of 70° is the best. The welding line energy, weld depth, weld width, and depth-to-width ratio in different wire feeding speeds and welding speeds are shown in Table 2, in which the welding line energy and depth-to-width ratio are calculated according to Formulas (1) and (2).

When the welding speed is 500 mm/min, the single factor method is used to study the variation of weld forming parameters in the wire feeding speed of 3 m/min~5 m/min. The results show that within the parameter range of wire feeding speed from 3 m/min to 5 m/min, the weld forming effect is best at 4 m/min. When the wire feeding speed increases from 3 m/min to 5 m/min, the welding current and welding voltage rise accordingly, and the welding line energy increases from 1345 J/cm to 2263 J/cm. The weld depth and width are increased with the rise in welding line energy. When the wire feeding speed is 5 m/min, the welding depth-to-width ratio is the best. However, the line energy in the wire feeding speed of 5 m/min is too fast to manufacture welded joints without burn-through defects. The forming effect in the wire feeding speed of 5 m/min is not ideal. Therefore, based on the wire feeding speed of 4 m/min, the next step is to study the influence of the welding speed in the range of 350 mm/min to 600 mm/min. The welding line energy decreases from 2533 J/cm to 1478 J/cm when the welding speed increases from 350 mm/min to 600 mm/min. The welding depth-to-width ratio decreases from 0.22 to 0.07. When the welding speed is higher than 350 mm/min, the forming effect of welded joints is well. There are no obvious welding defects on the surface. Therefore, penetration and burning loss are manifested when the welded joint in the wire feeding speed of 5 m/min.

## 4. Discussion

### 4.1. Microstructures of HAZ in Different Wire Feeding Speeds

**Figure 7:**
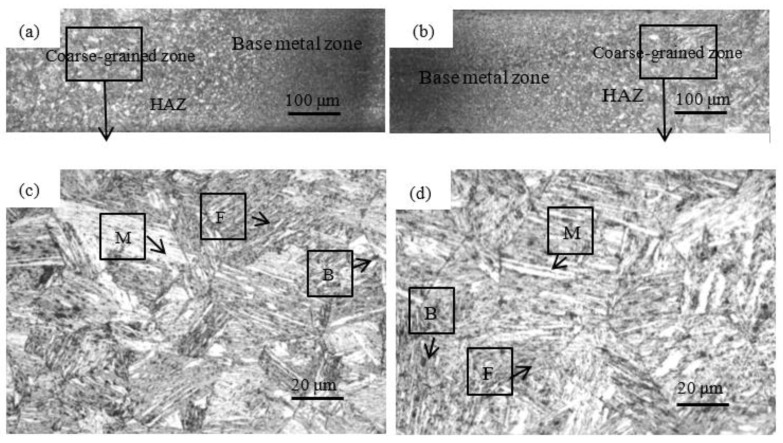
Microstructures of welded joints in different wire feeding speeds: (**a**) HAZ in the wire feeding speed of 3 m/min, (**b**) HAZ in the wire feeding speed of 5 m/min, (**c**) the coarse-grained zone in the wire feeding speed of 3 m/min, (**d**) the coarse-grained zone in the wire feeding speed of 5 m/min.

In the CMT + P mode, the welding speed of 500 mm/min is controlled and the effects of welding speed on the microstructures are shown in Figure 7. The HAZ is only a tiny area in the welded joint. However, failure of general materials occurs in this area. It is of practical significance to study the microstructures and grain size of the HAZ in welded joints. The HAZ of welded joints is divided into a complete phase transition zone and an incomplete phase transition zone according to the degree of the phase transition. The coarse-grained zone in the HAZ is a complete phase transition zone, but the grain size of the coarse-grained zone is larger than the other zone. Thus, the coarse-grained size is one of the reasons for the failure. Figure 7a,b, respectively, show the distribution of the HAZ at the wire feeding speed of 3 m/min and 5 m/min. The distribution range of the HAZ in the wire feeding speed of 5 m/min is more expansive than that in the wire feeding speed of 3 m/min. The heat input is increased with the rise in wire feeding speed, which leads to a broader HAZ range. Compared with the conventional CMT technology, the pulse can amplify the peak current to strengthen the penetration of the welder. Therefore, the heat input is reduced by increasing the welding speed to increase the grain size. At the same time, production efficiency is also improved.

As shown in Figure 7c,d, the microstructures of the coarse-grained zone are martensite and ferrite. The temperature of the melting pool exceeds the temperature of Ac3, which results in the dendrite remelting of the welded metal. The original microstructures are crystallized to form martensitic after rapid supercooling, which is consistent with the research [36] that adopts the laser to weld the DP980 dual-phase steel. However, the difference is that the DP980 dual-phase steel welded by the CMT + P technology also generates a small amount of bainite. The heat input of the CMT + P technology results in the formation of bainite after a small amount of remelted austenite through shear transformation and short-range diffusion. The grain size of the coarse-grained zone was measured by the “intercept method” in GB/T 6394-2017. When the wire feeding speed is 3 m/min, 4 m/min, and 5 m/min, the grain size of the coarse-grained zone is 31.90 μm, 39.84 μm, and 50.93 μm. It is found that the grain size is directly proportional to the wire feeding speed. The faster the wire feeding speed is, the higher the heat input per unit time is. Therefore, the more sufficient the grain growth time is, the coarser the grain size in the coarse-grained zone is.

### 4.2. Microstructures of HAZ in Different Welding Speeds

**Figure 8:**
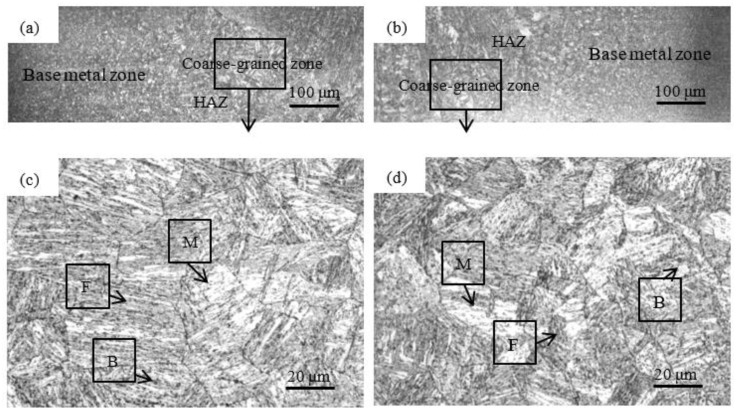
Microstructures of welded joints in different welding speeds: (**a**) HAZ in welding speed of 350 mm/min, (**b**) HAZ in welding speed of 600 mm/min, (**c**) the coarse-grained zone in welding speed of 350 mm/min, (**d**) the coarse-grained zone in welding speed of 600 mm/min.

**Figure 9:**
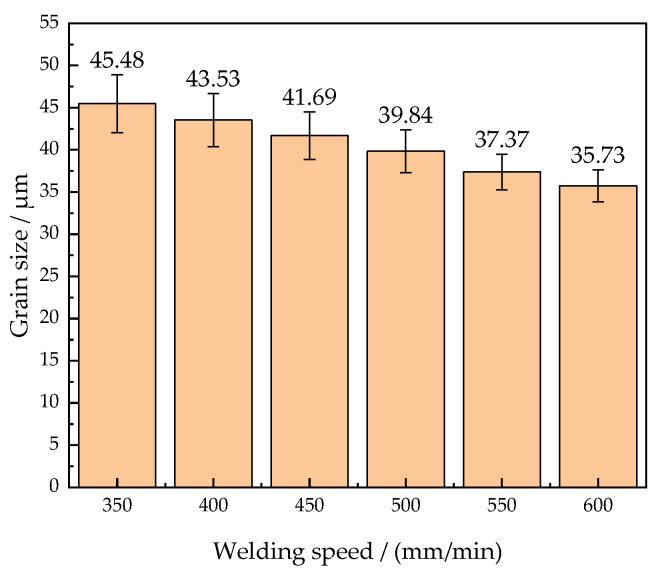
Grain size of the coarse-grained zone in different welding speeds.

In the CMT + P mode, the wire feeding speed of 4 m/min is controlled and the effects of welding speed on the microstructures are shown in Figure 8. The range of the HAZ in the welding speed of 600 mm/min is narrower than that in the welding speed of 350 mm/min. The heat input decreases accordingly to narrow the HAZ range. The temperature input into the HAZ also reaches the temperature of Ac3. The coarse-grained zone is composed of martensite, ferrite, and a small amount of bainite. When the welding speed increases from 350 mm/min to 600 mm/min, the martensite grain size in the coarse-grained zone decreases from 45.48 μm to 35.73 μm in Figure 9. It indicates that the grain size is inversely proportional to the welding speed. Due to the reduction of the heat input, it leads to the shortening of the grain holding time. The martensitic grains have no time to grow under the rapid cooling, which results in the formation of refined martensitic grains. Therefore, the influence of welding speed on the grain size is significant. The grain size of the coarse-grained zone is effectively refined by increasing the welding speed in CMT + P mode. The total amount of the heat input is decreased by increasing the welding speed. The pulse plays a role in amplifying the peak current and optimizing the welding arc. With the decrease of the heat input, the relative undercooling rate increases. The nucleation rate of microstructures in the coarse-grained zone increases and its growth rate slows down. Therefore, the grains of the coarse-grained zone are refined to improve the performance of the welded joints.

### 4.3. Microhardness of Welded Joints in Different Welding Parameters

**Figure 10:**
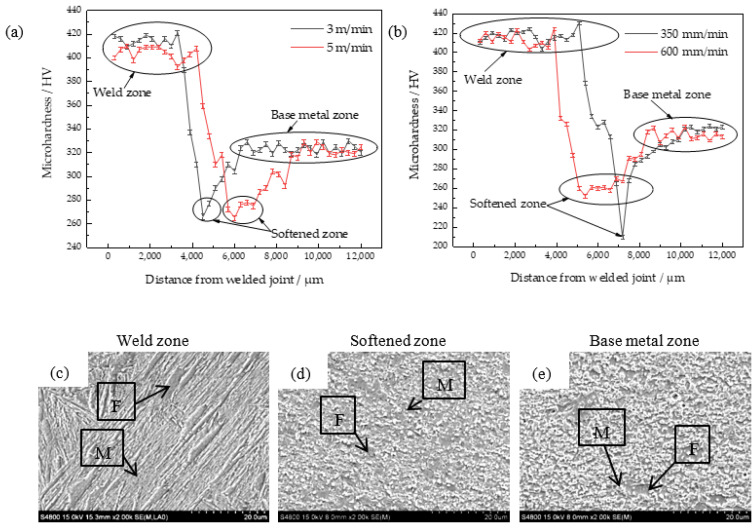
Microhardness and microstructures of welded joints: (**a**) microhardness in different wire feeding speeds, (**b**) microhardness in different welding speeds, (**c**) microstructure of the weld zone, (**d**) microstructure of the softened zone, (**e**) microstructure of the base metal zone.

The microhardness and microstructures of the welded joints under different welding speeds are shown in Figure 10. In addition to the coarse-grained zone analyzed above, there is also an incomplete phase transition zone called the softened zone in the HAZ. The welding temperature in the softened zone is between Ac1 and Ac3, which is the ferrite transformation condition. However, the softened zone is far from the welding heat source. Due to the unstable heat input, the size and distribution of the ferrite structure are not uniform. Thus, the comprehensive mechanical properties of the softened zone are poor.

Figure 10a shows that the distance of the softened zone in the wire feeding speed of 5 m/min is wider than that in the wire feeding speed of 3 m/min. The wire feeding speed is too fast, which results in a high total heat input and a sizeable thermal diffusion range. The HAZ deviates from the maximum temperature of the weld zone and the cooling speed is gradually accelerated. However, the temperature in this process is still higher than that of Ac1 for ferrite precipitation. Thus, more ferrite is formed in the softened zone.

Similarly, the heat input per unit of time is stable when the wire feeding speed is constant. The faster the welding speed, the shorter the residence time of the welding torch. Thus, the faster the undercooling rate, the more prominent the softened zone. As shown in Figure 10b, the welded joint in the welding speed of 350 mm/min has serious welding penetration. Due to the excessively high welding heat input, the microhardness in the softened zone decreases sharply. As the welding speed increases to 600 mm/min, the microhardness in the softened zone tends to be stable. Combined with the analysis of the line energy in Section 3.2, the welding line energy changes by 918 J/cm with the evolution of the wire feeding speed. At the same time, the line energy changes by 1055 J/cm with the evolution of the welding speed. It shows that the welding speed is the main factor affecting the heat input.

Figure 10a,b show that the microhardness of the welded joints is distributed in a high-low-medium trend. The high hardness area is the weld zone, which is formed by the filler. Because of the steady heat input, the microhardness in the weld zone is the highest. The average microhardness in the area is 420 HV and up to 430 HV. The weld zone composed of the lath martensite is shown in Figure 10c. Because the lath martensite is harder than the island-like martensite, its anisotropic has a stronger ability to hinder the movement of dislocations. Hence, the weld zone of the welded joint has a high microhardness.

The low microhardness area is the softened zone. The unstable martensite in the base metal is dissolved to undergo austenite transformation by heating cycles. After supercooling, parts of the new martensite are formed from the austenite. At the same time, the ferrite precipitates from another part of austenite. Due to the reduction of the original martensite, the microhardness of the softened zone decreases. The average microhardness in the softened zone is 250 HV. The softened zone composed of ferrite and island-like martensite is shown in Figure 10d. Part of the martensite in the base metal is transformed into ferrite after a short austenitizing process of heating, holding, and cooling. The remaining austenite is rapidly undercooled and transformed into the new martensite. Due to the enrichment of the ferrite in the softened zone, the microhardness drops sharply with the reduction of the martensite. It is the main reason for the failure of the welded joints.

The medium microhardness area is the base metal. The average microhardness is 320 HV, which is not strongly affected by the heat input. The base metal zone composed of ferrite and martensite is shown in Figure 10e. The martensite grain size in the base metal zone is larger than that of the softened zone, while the ferrite structure in the base metal zone is relatively reduced. It makes the base metal have higher hardness than the softened zone.

In conclusion, the expansion of the softened zone can be avoided by reducing the heat input. If the softened zone is reduced, the ferrite content in the softened zone will be decreased. That is, the microhardness can be improved by reducing the wire feeding speed or increasing the welding speed appropriately.

### 4.4. Tensile Strength of Welded Joints in Different Welding Parameters

**Figure 11:**
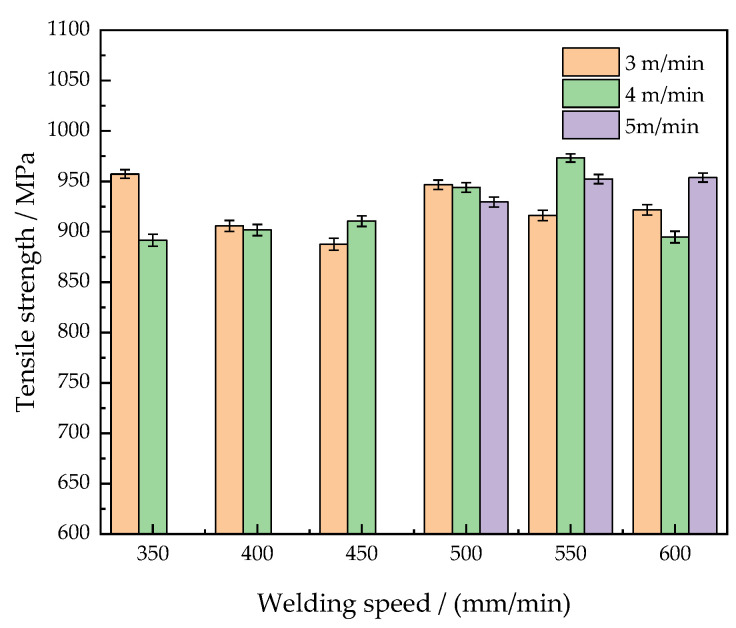
Tensile strength of welded joints in different welding parameters.

Tensile strength of welded joints under different welding parameters is shown in Figure 11. It shows that the wire feeding speed of 4 m/min and the welding speed of 550 mm/min are the optimum process parameters. The optimum tensile strength is 973 MPa. Referring to Table 2 above, the line energy is 1612 J/cm at this time. The line energy in the optimum process parameters is reduced by 921 J/cm compared to that in the welding speed of 350 mm/min, and the tensile strength in the optimum process parameters is increased by 9.15%. With the increase of the welding speed, the weld depth and weld width are respectively reduced by 1078 μm and 1489 μm. It can be observed that the reduction distance of the weld width is greater than that of the weld depth. With the increase of the welding speed, the welding heat input decreases in the welding process. However, the weld depth is increased by the action of the pulse when the weld width is 5279 μm. The weld width in the optimum process parameters is lower than that in the low welding speed, which indicates that the HAZ width is smaller. At the time, the average grain size of the coarse-grained zone in the HAZ is tiny, which is 37.37 μm.

When the welding speed increases to 600 mm/min at the wire feeding speed of 4 m/min, the tensile strength of the welded joint suddenly decreases to 895 MPa. Because the welding speed is too fast to approach the optimal line energy, this results in the narrowing of the weld bead and the poor bonding strength of the welded joint. However, when the welding speed exceeds 600 mm/min, the welded joints have obvious welding defects of incomplete fusion.

All the tensile strength of the welded joints under different welding parameters in Figure 11 is higher than 85% of the tensile strength of the base metals, which are qualified welding products. It indicates that welded joints with excellent tensile properties can be produced by the CMT + P welding technology. The average tensile strength of the welded joints is 925 MPa under different welding parameters. The tensile strength of the dual-phase steel welded by the CMT + P welding technology is 1.4 times higher than that of the DP1000 dual-phase steel welded by the electron beam welding technology [15]. Because the CMT + P welding technology utilizes the arc as the heat source, it has the advantages of a stable welding arc, low heat input, and no spatter in the welding process. Moreover, the pulse current can improve the welding heat input and arc stability. Increasing the welding speed is a good idea to neutralize the increase of the heat input, which not only optimizes the mechanical properties of welded joints but also improves the welding efficiency.

In conclusion, adopting the CMT + P technology to weld the DP980 dual-phase steel can obtain welded joints with excellent mechanical properties. The pulse enhances the stability of the arc, which is suitable for high-efficiency welding of thin plate materials. Taking the optimal tensile strength as standard, the wire feeding speed of 4 m/min and the welding speed of 550 mm/min are the optimal welding parameters. Its welding line energy is about 1612 J/cm.

**Figure 12:**
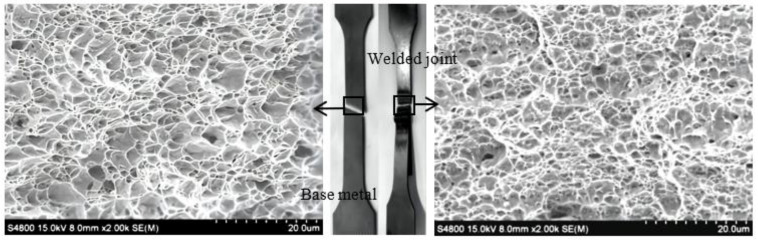
Fracture locations and morphology of the base metal and welded joint.

The fracture locations of the base metal and welded joint are shown in Figure 12. The fracture of the base metal occurs along the direction of the ferrite microcracks, and the fracture morphology is relatively flat. The fracture of the welded joint mainly occurs in the softened zone. Both of them are gray-black, cup-cone-shaped, and have no metallic luster. However, the fracture of the welded joint has a more significant tear ridge.

Compared with the ductile ferrite, the hard martensite has a stronger capacity to resist fracture and is less prone to fracture in the weld zone. Contrasted with the uniformly distributed ferrite in the base metal, the ferrite is enriched in the softened zone of the welded joint. The ferrite occurred in the softened zone consists of the ferrite without phase transformation and a small amount of the ferrite formed after austenite recrystallization. The excessive ferrite results in plastic deformation after stretching and the ductile fracture finally occurs in the HAZ.

According to the SEM morphology of the base metal, the microstructures of the fracture are evenly distributed. There are a large number of tensile equiaxed dimples in the fracture. Under the conditions of the unidirectional tensile, a three-dimensional stress concentration of the ferrite grain is caused, and microcracks are generated at the phase boundary between the ferrite and the martensitic [37]. As the cracks expand and merge, tiny dimples appear. The formation of fracture dimples indicates that the fracture mode of the base metal is microporous aggregation fracture. There are also dimples in the fracture of the welded joint. However, the size of the dimples in the welded joint is smaller than that of the base metal. It means that the welded joint has no larger buffer space than the base metal when plastic deformation occurs. Therefore, the welded joint has less ability to resist ductile fracture than the base metal.

## 5. Conclusions

(1) By using the CMT + P 70° lap welding method and the ER120S-G wire to weld the DP980 dual-phase steel with the thickness of 1.2 mm, the microstructures and properties of the welded joints are excellent. In the welded joints, Si and Ni are the main elements added. The martensite, ferrite, and a little bainite are the primary microstructures. By increasing the wire feeding speed from 3 m/min to 5 m/min, the average grain size of the coarse-grained zone increases from 31.90 μm to 50.93 μm. By increasing the welding speed from 350 mm/min to 600 mm/min, the average grain size of the coarse-grained zone decreases from 45.48 μm to 35.73 μm.
(2) The average microhardness of the weld zone is 420 HV, which is significantly higher than that of the base metal. The peak microhardness is 430 HV. The minimum microhardness occurred in the softened zone of the HAZ is 250 HV. The welding speed is the main factor affecting the range of the softened zone. By increasing the wire feeding speed or decreasing the welding speed, the range of the softened zone becomes wider.
(3) The wire feeding speed of 4 m/min and the welding speed of 550 mm/min are the optimum process parameters, when the tensile strength of the welded joint is 973 MPa. The tensile fracture is located in the softened zone and the fracture mode is a ductile fracture. In the CMT + P process, the effect of process parameters on the welding depth is greater than the welding width. The welding line energy for optimal process parameters is 1612 J/cm, the weld depth is 0.437 mm, and the weld width is 5.279 mm.

## Figures and Tables

**Table 1:** Chemical compositions of DP980 dual-phase steel and ER120S-G welding wire (mass fraction/%).

Materials	C	Si	Mn	P	S	Nb	Ti	Mo	B	N	Als	Cr	Ni
DP980	0.091	0.307	2.464	0.012	0.001	0.016	0.049	0.153	0.002	0.005	0.047	-	-
ER120S-G	0.080	0.770	1.780	0.008	0.002	-	-	0.580	-	-	-	0.340	2.310

**Table 2:** Welding line energy and depth-to-width ratio in different welding process parameters.

Wire Feeding Speed/m/min	Welding Speed/mm/min	Welding Current/A	Welding Voltage/V	Line Energy/J/cm	Weld Depth ^1^ μm	Weld Width ^1^/μm	Depth-to-Width Ratio
3	500	59	19	1345	515	4323	0.11
4	500	75	19.7	1773	708	5549	0.12
5	500	92	20.5	2263	1303	6512	0.20
4	350	75	19.7	2533	1515	6768	0.22
4	400	75	19.7	2216	837	6325	0.13
4	450	75	19.7	1970	799	5900	0.13
4	550	75	19.7	1612	437	5279	0.08
4	600	75	19.7	1478	350	4711	0.07

^1^ The average confidence interval of the weld depth is ±10 μm and that of the weld width is ±50 μm.

## Data Availability

Not applicable.
